# A holistic approach for quantifying the value of public health programs: social return on investment (SROI) analysis of a mobile clinic as an example

**DOI:** 10.3389/fpubh.2026.1650485

**Published:** 2026-02-25

**Authors:** Shubha Kumar, Aaron Mallett, Sara Olsen, Anne Coleman

**Affiliations:** 1 Department of Population and Public Health Sciences, University of Southern California, Los Angeles, CA, United States; 2 SVT Group, Milpitas, CA, United States; 3 Jules Stein Eye Institute, University of California, Los Angeles, Los Angeles, CA, United States

**Keywords:** public health programs, social return on investment, economic evaluation, mobile clinic, impact

## Abstract

**Introduction:**

Public health programs often face challenges in demonstrating their full impact through traditional evaluation methods or economic approaches such as cost–benefit analysis or cost-effectiveness analysis.

**Methods:**

Using a social return on investment (SROI) analysis approach, we evaluated the broad social and economic impact and value created by UCLA Mobile Eye Clinic’s Pre-School Vision Program. This program screened 90,000 pre-school children in underserved areas of Los Angeles County between 2012 and 2017, providing glasses and referrals as needed. The evaluation study employed mixed methods consistent with the process of SROI analysis.

**Results:**

Results indicate the strong positive impact and value of the program, generating approximately $40 of value in the community for every $1 invested. Various outcomes were identified by key stakeholders, with the most valuable being improved quality of life for pre-school children who received glasses. Other highly valued outcomes included increased confidence and self-esteem of the children, their decreased dependency on others, and their increased responsibility.

**Discussion:**

These findings suggest that increased provision of mobile clinics for communities including children living in underserved areas could produce significant value. Findings also demonstrate the relevance of SROI for evaluating and quantifying the impacts of public health programs more broadly.

## Introduction

As demand for public health resources often outweighs supply, public health officials and practitioners are increasingly called upon to measure and demonstrate the impact, including value-for-money, of interventions (1). Funders increasingly seek optimization between the financial inputs they provide and the health outcomes that are achieved as a result of funding (2–4). However, quantifying value-for-money of investments in health has remained a challenging endeavor, partly due to the sheer scale of the impacts that can arise from health interventions (5, 6). Traditional economic evaluation methods used in public health such as cost-effectiveness analysis (CEA) or cost benefit analysis (CBA) often fall short in various ways, whether it be their suitability, implementation challenges, lack of comprehensiveness, or other factors (7, 8).

Social return on investment (SROI) analysis is a methodology that has been advanced as a more pragmatic and holistic framework to assess impact of interventions that measures the value generated by an intervention by considering its broader impact on all stakeholders in the local area in which the intervention is implemented. However, its application to public health programs is not yet mainstream (1, 5, 9, 10). SROI analysis is a process for understanding, measuring, and reporting on the social, environmental, and economic value created by an organization, program, or policy (11). It is a mixed methods approach employing both quantitative and qualitative techniques, with an emphasis on stakeholder engagement throughout the process. Compared with CEA which typically measures the value of improved health resulting from an intervention in natural units (e.g., lives saved or cases averted), SROI measures the value of the range of outcomes which result from an intervention, including health, social, economic and environmental outcomes as relevant, and in monetary terms (9). And while CBA also tends to consider a wider range of outcomes and in monetary terms, outcomes in CBA have typically been identified from the perspective of the government or analyst, whereas in SROI outcomes are identified by key stakeholders who are affected by or affect the intervention (9). Furthermore, CBA often lists any benefits that cannot be easily monetized and explains why so, whereas SROI analyses usually deliberately attempt to leverage financial proxies to estimate monetary value of outcomes that cannot be easily monetized (e.g., empowerment, self-confidence) to the extent feasible in SROI’s attempt to measure and account for the full value of an intervention (9). SROI is based on a set of principles, articulated by Social Value International (SVI) (the standard bearer for SROI methodology and assurance), which underlie how SROI should be applied (11, 12).

Similar to CBA, a key result of SROI analysis is the determination of the SROI ratio, which compares the value of the benefits with the investment. For example, an SROI of 3:1 indicates that for every $1 invested in an intervention, it delivers $3 in value.

This article presents the SROI methodology as applied to a public health program and lessons learned from this work, using a mobile pre-school vision program as an illustrative example. Importantly, the SROI analysis process, which can be applied to any public health program, not only produces a measure of impact, but also key insights with respect to strengths of and opportunities for program implementation. The broader implications of this approach to further impact measurement in public health monitoring, evaluation and implementation efforts are explored in the discussion section.

## Methods

An evaluative retrospective social return on investment (SROI) analysis was conducted to evaluate the broader impacts of the Pre-School Vision Program implemented by the University of California Los Angeles Mobile Eye Clinic (UMEC) over a five-year period. UMEC is an intervention which visits various Southern California community locations annually to deliver free basic vision care (including glasses and referrals to specialists as needed) to pre-school aged children in underserved communities across Los Angeles County. Between 2012 and 2017, UMEC completed vision screenings for 90,000 preschool-aged children with funding support from First 5 LA.

Consistent with SVI guidelines, we followed the six stages of SROI analysis: establishing scope and identifying key stakeholders, mapping outcomes, evidencing outcomes and giving them a value, establishing impact, calculating the SROI ratio from the perspectives of key stakeholders, and reporting (see Table 1) (11–13).

**Table 1:** Overview of SROI methodology as applied in the case study.

Stage	Description	Application in the UMEC SROI evaluation
Stage 1	Establishing scope and identifying key stakeholders	Scope of analysis: 2012–2017, 4 LA County areas. Stakeholders: Preschool children, Parents, Pre-school staff, UMEC staff and volunteers, EyeDeal Optical (Glasses Supplier), First 5 LA (funder).
Stage 2	Mapping outcomes	Literature review to identify common outcomes of pediatric vision care programs. Stakeholder engagement via focus groups; Thematic analysis of focus group transcripts using NVivo.
Stage 3	Evidencing outcomes and giving them a value	Assess representative sample of children receiving glasses. Survey to determine the deadweight, outcome duration, quantity of outcome, drop-off, and attribution. Integrate data into impact map and identify financial proxies from literature.
Stage 4	Establishing impact	The impact of each outcome was calculated by multiplying the extrapolated quantity of individuals experiencing the outcome by its financial value, deadweight, attribution, and drop-off.
Stage 5	Calculating the SROI	Sum total value of outcomes over their duration. Compare to the total program budget (2012–2017). Conduct sensitivity analysis of SROI ratio.
Stage 6	Reporting, using and embedding	Report SROI results to UMEC and stakeholders. Discuss program strengths and opportunities for implementation improvements.

### Establishing scope and identifying key stakeholders

In Stage 1 of the SROI analysis, the scope of the analysis was determined through conversation between academic partners and UMEC leadership. It was agreed that the scope of the analysis would include measurement of outcomes experienced by key stakeholders between 2012 and 2017, and, in four areas of Los Angeles County where UMEC was implemented: West LA (Santa Monica), East LA (San Gabriel), Central LA (Downtown), and South LA (San Pedro). These areas were chosen for their geographic and community diversity. Next, key stakeholders of the program were identified as those people, organizations, or entities that contribute to or experience change as a result of the intervention. The key stakeholders identified were: pre-school children, their parents, pre-school staff including teachers and nurses, UMEC staff and volunteers, EyeDeal Optical (a glasses company who provided glasses for the program), and First 5 LA (funder). The majority of children who participated in the program were Latino (79%), followed by African-American (5%), Asian (5%), White/Caucasian (4%), and other race/ethnicity (5%). It was decided that all key stakeholders would be included and engaged in the SROI process via focus groups, except for First 5 LA given its priorities were understood by UMEC and embodied by the outcomes other stakeholders experienced.

### Mapping outcomes

In Stage 2, a two-fold process was undertaken to develop a theory of change – an articulation of how the activities are understood to cause changes over time for various affected stakeholders, and what those changes are understood to be. First, a literature review was conducted to identify the current state of evidence with respect to common outcomes of vision care programs for children. Findings of the literature review revealed a multitude of outcomes including improvements in quality of life, economic benefits, educational benefits, developmental benefits, psychosocial benefits, and others (14–19). One source identified during the literature review – the World Health Organization (WHO) model about investments in children’s health – was used to guide the development of the preliminary theory of change (20). The second part of this step included engaging with key stakeholders via a series of focus groups to understand from their perspectives what were the key outcomes of the program from their experiences. A total of 7 focus groups were conducted with key stakeholder groups including parents (who spoke on behalf of both themselves and their pre-school aged children), school staff, and program delivery staff from UMEC and EyeDeal Optical. Approximately 10–15 participants participated in each focus group.

**Figure 1:**
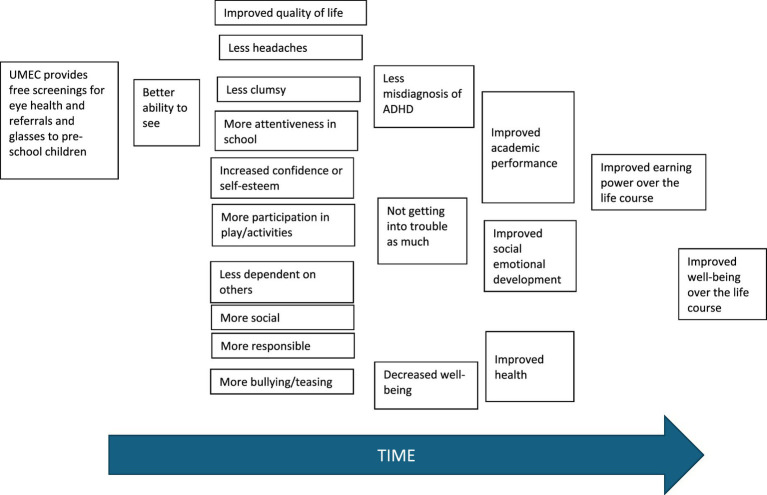
Theory of change for pre-school children who receive glasses from UMEC.

Thematic analysis of focus group transcripts was conducted using NVivo to identify the key outcomes experienced by stakeholders and how they described them in their own words (indicators). These outcomes were then mapped into a theory of change for the intervention (see Figure 1 for the theory of change for one key stakeholder group – pre-school children who were screened, identified as needing glasses and received glasses from UMEC). In addition, analysis of the focus groups validated that there were no other key stakeholders who had not been considered and to move on to next steps.

### Evidencing outcomes and giving them a value

After focus groups findings were analyzed, further evidence of outcomes was collected from a representative sample size of the stakeholder group most materially affected – the pre-school children who were screened with a priority focus on those identified as needing glasses, and received glasses through the program – to identify further information about the outcomes they experienced. A total of 8,105 children out of the 90,000 children that were screened over the 5-year period fell into this category. Other stakeholder groups (i.e., parents, school staff, program staff) were not engaged beyond the focus groups regarding outcomes they experienced either because a representative sample was already engaged, their outcomes were not as material, or due to resource constraints. In this round of data collection, a survey instrument was developed based on findings from the focus groups with parents of the children who were screened to gain a general understanding of their experiences, and for the children who received glasses from UMEC, specifically to determine key factors to enable the SROI calculation: (a) the number of children experiencing relevant outcomes as identified during the focus groups (quantity), (b) the relative importance to children and parents of those outcomes, (c) the amount of outcomes that would have been experienced even in the absence of the program (“deadweight”), (d) how long outcomes lasted (duration), (e) the deterioration of outcomes over time (“drop-off”), and (f) how much of the outcomes experienced were caused by the contribution of other organizations or people (“attribution”). A simple random sampling approach was employed from a list of participants, which included phone contact information largely for those who were screened and had received glasses from UMEC as well as contact information for some children who had been screened but not received glasses from UMEC. A total of 203 survey interviews were completed with children’s parents or guardians by phone in English or Spanish based on respondent preference. Approximately 75% of respondents represented children who had been screened and received glasses through UMEC. The remaining 25% of the sample represented other pre-school children: (1) pre-school children who were screened and not diagnosed as needing a glasses prescription, (2) pre-school children who were screened, diagnosed as needing a glasses prescription, but for some reason did not receive glasses from UMEC, and (3) pre-school children who were screened, diagnosed as needing a glasses prescription, and obtained glasses through other providers. To note, the purpose of data collection with these latter sub-groups of preschool children was to understand their overall experiences and any feedback they may have, though data collection with these sub-groups was not as extensive nor was their data factored into the SROI calculation (see Appendix for survey instrument). Data from the survey interviews was then entered into Microsoft Excel and analyzed for summary statistics and to calculate an estimate of the extrapolated quantity of children who experienced each outcome based on the representative sample of the total population size of this group (Table 2). For preschool children who received glasses from UMEC, excel was further used to calculate estimates of deadweight, attribution and drop-off of outcomes from survey data.

**Table 2:** UMEC SROI survey results for preschool children who received glasses from the program.

Outcomes	Percent of survey respondents answered yes (or 5, 4, 3 on Likert scale)
Improved quality of life	88% (130/147)
More responsible	68% (79/116)
More attentiveness/better performance in school/grades	67% (98/147)
Increased self-confidence and/or self-esteem	63% (87/139)
Less dependent on others	58% (71/123)
More participation in play/activities	53% (77/145)
More bullying/teasing	13% (17/127)
Not getting into trouble as much	3% (4/121)

Relevant data on outcomes, quantity, deadweight, attribution, and drop-off from the surveys were then integrated into the preliminary impact map in Excel. The inclusion of deadweight, drop-off, and attribution in the impact map was critical so as not to overstate the impact of the program, as is consistent with SVI standards (21).

Next, to identify the monetary value of each outcome, a search of academic and gray literature was conducted to identify financial proxies of outcomes according to previous literature. The proxies that were deemed most relevant and credible for each outcome were then entered into the impact map and adjusted as needed (i.e., calculations were made to adjust for the market rate in the relevant locality, to adjust for a yearly value, etc.) (see Table 3).

**Table 3:** UMEC pre-school vision program impact map valuations (condensed version).

Stakeholder	Outcomes	Extrapolated quantity	Financial proxy	Monetary value	Source	Deadweight	Attribution	Drop-off	Impact
Pre-school children screened, diagnosed as needing glasses, and who received glasses	Improved quality of life	7,132	Valuation of Vision Related Quality of Life Losses for Children aged 0–17 (Value per year calculated based on only mild–moderate vision impairment QALYs for 0–17). Calculation: (QALY*$50 K for visual impairment/Prevalence mild + medium impairment).	$4,689	Wittenborn et al. (2013) (22)	9%	25%	100%	$22,825,410
	Not getting into trouble as much	243	Benchmark of avg. cost of therapy twice/month over 1 year (Value calculated based on market rate in locality).	$3,600	McGrath and Stevens (2017) (23); Lakhotia (2019) (24)	9%	25%	100%	$597,420
	More attentiveness/better performance in school/grades	5,430	Cost of tutoring/year (Value calculated based on market rate in locality).	$720	Ernst and Young, Eastern Media International, Eastern Charity Foundation (2021) (25)	9%	25%	100%	$2,668,474
	Increased self-confidence and/or self-esteem	5,106	Benchmark of avg. cost of therapy twice/month over 1 year (Value calculated based on market rate in locality).	$3,600	McGrath and Stevens (2017) (23); Lakhotia (2019) (24)	9%	25%	100%	$12,545,811
	More participation in play/activities	4,296	Benchmark of avg. cost of after school program in public school for 1 year (Value calculated based on market rate in locality).	$2,500	Lakhotia (2019) (24); Bellucci et al.	9%	25%	100%	$7,329,453
	Less dependent on others	4,701	Cost of anxiety related therapy + medication / self-confidence courses (Value calculated based on market rate in locality).	$3,600	McGrath and Stevens (2017) (23)	9%	25%	100%	$11,550,111
	More responsible	5,511	Benchmark of avg. cost of after school program in public school for 1 year (Value calculated based on market rate in locality).	$2,500	Lakhotia (2019) (24); Bellucci et al.	9%	25%	100%	$9,403,826
	More bullying/teasing	1,054	Benchmark of avg. cost of therapy twice/month for 1 year and 6 co-pays for dr. visits (Value calculated based on market rate in locality).	-$3,720	Lakhotia (2019) (24)	9%	25%	100%	-$2,675,112
	Avoided blindness	1	QALY (Value per year calculated based on Blindness QALYs for 0–17). Calculation: (QALY*$50 K for blindness/Prevalence blindness).	$13,858	Wittenborn et al. (2013) (22)	9%	25%	100%	$9,458
	Avoided other vision impairment	26	QALY (Value calculated based on Midpoint of QALYs per year for 0–17 blind ($13,858) and 0–17 mild–moderate ($4,688)).	$9,273	Wittenborn et al. (2013) (22)	9%	25%	100%	$162,825
Parents/Guardians	Parents received benefit of knowledge and feeling of relief of knowing child’s eye health status due to screening received	86,823	$ amount of (avoided) lost wages due to day off work (Value calculated based on market rate in locality).	$120	Market rate (based on minimum wage)	9%	25%	100%	$7,110,804
	Parents obtained affordable eye health screening for their child	86,823	Benchmark of avg. cost of an eye exam in the US (Value calculated based on market rate in locality).	$130	Ernst and Young, Eastern Media International, Eastern Charity Foundation (2021) (25)	9%	25%	100%	$7,703,371
	Increased awareness of eye health information	86,823	$ amount 1 visit with health educator (Value calculated based on market rate in locality).	$20	Market rate	9%	25%	100%	$1,185,134
Pre-school teachers	Increased awareness of eye health information	1,286	$ amount 1 visit with health educator (Value calculated based on market rate in locality).	$20	Market rate	9%	25%	100%	$17,554
	Increased job satisfaction	1,286	Hourly wage of teacher x number of school days/year (based on being willing to stay an extra hour per day) (Value calculated based on market rate in locality).	$2,700	Ernst and Young, Eastern Media International, Eastern Charity Foundation (2021) (25)	9%	25%	100%	$2,369,777
	Disruption to school activities due to UMEC screenings	1,286	Hourly wage of teacher*2 h (Value calculated based on market rate in locality).	-$30	Ernst and Young, Eastern Media International, Eastern Charity Foundation (2021) (25)	9%	25%	100%	-$26,331
School Nurses and Advocates	Increased job satisfaction	8	Hourly wage of school nurse x number of school days in year (based on being willing to stay an extra hour every day) (Value calculated based on market rate in locality).	$4,905	Ernst and Young, Eastern Media International, Eastern Charity Foundation (2021) (25)	9%	25%	100%	$26,781
UMEC Staff	Helped to feel part of the LA community	1	Cost of social class (i.e., Pilates class or other activity where there is adult integration) weekly over a year (Value calculated based on market rate in locality).	$2,500	Lakhotia (2019) (24)	9%	25%	100%	$1,706

### Establishing impact and calculating the SROI

Once all of the above factors were entered into the impact map, it enabled calculation of the SROI ratio and finalization of the impact map (see Table 3 for a condensed version). First, the ‘impact’ (as defined by SVI) of each outcome was calculated by multiplying the extrapolated quantity of individuals who experienced each outcome by that outcome’s financial value, deadweight, attribution and drop-off. The impact results indicate the value created (or destroyed) per year. Next the total value of all outcomes was summed together (including both the positive and negative values) to arrive at the total value created. Then the time period during which outcomes were experienced (including during the period of activity and beyond) were taken into account. The total value over this time period was then compared with the total amount of investment (i.e., the total investment was based on the total program budget from 2012 to 2017 as no additional in-kind resources were provided) to arrive at the SROI ratio.

Next, sensitivity analyses were conducted to test key assumptions or factors of the SROI model and how changes to these would influence the SROI ratio result. The sensitivity analysis tests conducted included: (a) changing the total monetary value of all outcomes to 50 and 150% of the base case (in case monetary values were over or under-estimated), (b) changing the deadweight to 25% for all outcomes (in case deadweight values were under-estimated), and (c) changing the value of one specific outcome (i.e., improved quality of life) which was largely driving the total value creation to 20, 50 and 150% of the base case (in case the value of this outcome was over or under-estimated). Results from these sensitivity tests allowed for the establishment of a robust range within which the SROI ratio falls.

### Reporting, using and embedding

In the last stage, SROI results (including both quantitative and qualitative) were reported to UMEC along with a plan for dissemination to other key stakeholders. Findings were discussed including both the strengths of and opportunities for program implementation, which were subsequently used by UMEC toward engagement with key stakeholders and future implementation.

### Methodological assumptions and limitations

Both for purposes of this study as well as broader application of the SROI methodology to public health programming in general, it is important to discuss the assumptions and limitations that were applied when conducting this SROI analysis.

First, with respect to valuation, after the specific outcomes of the program were identified, they were translated into monetary values using financial proxies. The financial proxies were based on techniques found in the literature, other SROI analyses, or benchmarks in instances where other sources could not be found (22–25). In some cases, it was decided not to monetize outcomes as it was unclear whether the data collected on those outcomes was reliable and in one case a credible approach to monetization could not be determined. While these outcomes were included in the full version of the impact map, they were not monetized or included in the value calculations.

Second, four main assumptions were considered when establishing program impact and developing the SROI model: attribution, deadweight, displacement, and drop-off. While conducting a randomized control trial (RCT) is considered the gold standard to identify estimates of such factors, RCTs are not always feasible nor appropriate to conduct. As SROI methodology was developed as a pragmatic approach to be used by practitioners, importance is placed on recognizing these factors exist and trying to ascertain a best estimate for these factors as feasible, such as from previous literature or from the perspectives of those experiencing outcomes. This study leveraged survey interview results to deduce estimates for attribution and deadweight across all outcomes. Given the length of the survey, it was not feasible to collect attribution and deadweight data at the level of each individual outcome and therefore a collective approach was employed to establish average values for attribution and deadweight across outcomes. In terms of displacement, this occurs when an intervention’s positive impact on a group effectively negates or reduces a positive outcome that would have occurred for another group, either within or outside the intervention. A classic example of displacement is that of a neighborhood watch program that reduces crime in one area but results in an increase in crime in an adjacent neighborhood (13). In this study, it was assumed that displacement was zero.

With respect to drop-off, there is recognition that following the end of a program, benefit is usually still created for some time into the future. However, the amount of outcome value gained is likely to be less or will be more influenced by other factors, so a drop-off percentage on a year-on-year basis needs to be determined. In this context, which is often the case in public health, some outcomes may play out over decades, such as improved quality of life or increased earning potential, while others may be short-lived, such as bullying. Given this variability, and the fact that the UMEC program did not have the capacity for ongoing evaluation of longer-term outcomes, it was assumed that drop-off was 100% after the first year as a conservative simplifying estimate. Relatedly, a discount rate was not applied.

Third, assumptions were made for specific outputs for which data were not available. For example, the number of teachers and the number of nurses were estimated based on state ratios for teachers to students and nurses to students, respectively.

Given such assumptions and limitations of the study, and as per the SROI analysis process, conducting the sensitivity analyses were important to test the robustness of the SROI ratio.

Fourth, it is worth noting here one of the general limitations of SROI analysis—SROI ratios between different programs with different stakeholders and different contexts should not be compared. In this study, evaluators applied the internationally-recognized and standardized approach to SROI (as set out by SVI) which recognizes there is inherent subjectivity in stakeholder’s perceptions and how they may describe the outcomes they experienced. To minimize the subjectivity of results, data was collected from a representative sample size of the population. In terms of the monetary value of outcomes found in the literature, these were based on the most relevant evidence that could be identified at the time, and as the literature develops, there will likely be more or different evidence to draw from. Collecting data from stakeholders, conducting the valuation based on available data and evidence, conducting sensitivity analysis including for monetary valuations as relevant, and presenting this information and any sources and assumptions transparently, are all connected to the key principles underlying how SROI analysis should be conducted. If one were to attempt to re-produce the methodology for the same intervention, one could do so, or one could update it based on available evidence at the time conducted, which may or may not lead to similar results. The primary value of SROI analysis lies not in comparing results of different interventions, rather in comparing the results of the same intervention over time as it responds to evaluation findings and subsequent changes in value creation over time.

## Results

The evaluation found the SROI ratio of this program to be approximately $40:$1, that is, for every $1 invested in the program, approximately $40 in social value was created in the community. Results of the sensitivity analyses conducted suggest that, at the very least the program would have an SROI of approximately $19:$1 and at most an SROI of $59:$1 (see Table 4). The SROI ratio and sensitivity range strongly indicate the value of UMEC exceeds the investment. Of the total impact, the most value was created for pre-school children who were screened, diagnosed as needing glasses, and who received glasses (78%), followed by parents/guardians (19%), followed by schoolteachers (3%) and by other key stakeholders (3%). This distribution of impact is consistent with the primary beneficiaries of the intervention.

**Table 4:** SROI sensitivity analysis results.

Assumption tested	Test performed	Initial SROI	New SROI	Change
Monetized value of all outcomes	50% of base case	39.71	18.86	−20.85
Monetized value of all outcomes	150% of base case	39.71	58.57	+18.86
Deadweight	Increased deadweight to 25% across all outcomes	39.71	36.37	−3.34
Monetized value of outcome: improved quality of life	20% of base case	39.71	29.96	−9.75
Monetized value of outcome: improved quality of life	50% of base case	39.71	33.24	−6.47
Monetized value of outcome: improved quality of life	150% of base case	39.71	44.19	+4.48

For pre-school children who received glasses from UMEC, 16 outcomes were identified, of which 10 were monetized (the remaining 6 outcomes were not included in the value calculation as it was unclear whether the data collected on those outcomes was reliable). During survey interviews with parents, the following outcomes experienced by children were cited most frequently: improved quality of life, increased attentiveness and/or performance in school, and improved confidence and/or self-esteem (see Table 2). Of all the outcomes experienced by the children that were monetized, the three that generated the most value were (a) improved quality of life, (b) increased confidence and/or self-esteem, and (c) less dependency on others. There were several additional outcomes which generated positive value (refer to Table 3). To note, there was also an outcome experienced by the children—increased bullying or teasing (as a result of wearing glasses) – which generated negative value. It is also worth noting that pre-school children who were screened but did not require nor receive glasses also experienced a benefit from the intervention in terms of knowing their health status, though this benefit was not included in the SROI valuation.

For parents/guardians, all outcomes experienced were of a positive value. These outcomes included: (a) parents received benefit of knowledge and feeling of relief of knowing their child’s eye health status due to screening received, (b) parents obtained affordable eye health screening for their child, and (c) increased awareness of eye health information. All of these outcomes were monetized.

For schoolteachers, the key outcomes experienced were: (a) increased awareness of eye health information, (b) increased job satisfaction, and, on the negative side, (c) a disruption to school activities due to the UMEC screenings. All of these were monetized.

For school nurses and family service advocates, one key outcome was experienced: increased job satisfaction, and this was monetized. Of note, school teachers, school nurses and family advocates stated that they felt their job satisfaction increased given the impact they have not only on overall health and development, or scholastic improvement of the child, but also specifically on the child’s eye health as a result of supporting children to be screened and reminding them to wear glasses.

For UMEC staff, two outcomes of program involvement were identified: (a) increased job satisfaction and (b) helping to feel part of the Los Angeles community. The first outcome was not monetized as the activities performed were a central part of their jobs so it was considered that effectively their pay reflects this value and income of implementing staff members is not considered an outcome in SVI methodology. With respect to the latter outcome, one staff member described having moved to Los Angeles recently from another city and that the program helped him feel more connected with his new community. This outcome was monetized.

For EyeDeal Optical staff, one key outcome was experienced: increased job satisfaction. The co-owner of the company shared that participation in this program motivated him to start a similar non-profit organization in his home country of Guatemala to support children’s eye health. This outcome was not monetized as it would have required additional research with Eyedeal Optical staff that was out of scope of this analysis.

## Discussion

Results suggest a very positive impact of the program, with benefits generated for various key stakeholders including pre-school children, parents/guardians, schoolteachers, school nurses and family service advocates, EyeDeal Optical, and UMEC staff. The sensitivity range of the SROI ratio determined in this study is consistent with broader findings on return on investment studies of early childhood and public health interventions, and that an ounce of prevention is worth a pound of cure (1). While $40:$1 may seem relatively high, results are consistent with previous studies which have generally found vision care for preschoolers to be a beneficial and cost-effective investment, including one study which found a benefit-to-cost ratio of visual acuity screening among preschoolers as high as $162:$1 (18, 26, 27). That data was collected from a representative sample size and that SROI results remained robustly positive under sensitivity analysis testing in this study contribute to the quality of the study and its results. As the sensitivity analysis range explicitly acknowledges the inherent uncertainties and judgments involved in an SROI calculation, and that in this case, the low end of the relatively wide range was still highly positive ($19:$1), this range provides essential context for informed decision-making. In addition, evaluators employed a conservative approach to valuation, for example, in only including those material outcomes for which reliable data was collected and in only accounting for outcomes as lasting for 1 year (even though it is very likely their benefits accrue for longer than a year, and in some cases over the life span), both of which demonstrate the application of the SROI principle to not over claim results in this study.

Focus groups and survey interviews highlighted strengths of program implementation including removing barriers to access to care through convenience of the school location, timings and free services and glasses. In addition, parents and school staff cited the benefits of increased health awareness not only for the children, but for the parents and siblings as well. Focus groups also highlighted some recommendations to improve the program implementation including the ability for children to choose different styles of glasses which could enhance self-image and improve compliance, particularly for children who experienced bullying. Bullying has been documented in other similar interventions and key strategies emphasize the importance of educating parents and teachers on the importance of wearing glasses and their critical role in encouraging children to wear them (28–31). Additional opportunities identified during focus groups for future implementation included increased frequency of screenings and hours for parents to interact with UMEC, offering additional replacements beyond the one-time glasses replacement, and faster turnaround time between screenings and glasses delivery. In addition, focus groups revealed a clear need to collaborate with local pediatricians on eye care for this population to improve understanding of eye health, diagnoses, and adherence to wearing glasses for children. Focus groups also identified an opportunity for future research with respect to the prevalence of misdiagnosis of ADHD or other behavioral diagnoses due to undiagnosed vision impairment, as experienced by some of the children. In summary, this study demonstrated that investments in the UMEC Pre-School Vision Program are valuable for various stakeholders in many ways, and that the developmental and educational benefits set children up for long term success and increase their quality of life both immediately and throughout the lifespan.

Beyond evaluation of the program itself, several broader lessons were learned as a result of applying the SROI methodology to this public health intervention. Compared to standard economic evaluation approaches, using an SROI approach presented key advantages. First, unlike traditional return on investment analysis, this research took an inductive approach. It did not test hypotheses formulated ex-ante, but involved stakeholders in identifying the changes they experienced, and the outcomes (both positive and negative, intended and unintended) which ought to be included in the analysis. This allowed the incorporation of those outcomes which were deemed most important to stakeholders themselves, as opposed to pre-defining what ought to be important from the standpoint of others such as program donors or managers. Secondly, the SROI analysis did not incorporate solely what was easy to quantify and value (so-called ‘hard’ economic or health impacts). Rather, it also considered key ‘soft’ impacts which have traditionally been excluded from return on investment analysis (e.g., confidence and social developments of children). Critically, this study found that ‘softer’ (or less tangible) impacts were extremely valuable to stakeholders, often more so than ‘hard’ ones. Of course, SROI methodology also has its challenges. Similar to cost–benefit analysis, the lack of standard financial proxies for outcomes is one of the biggest challenges in SROI (32, 33). Furthermore, depending on the methods used, there may be a high degree of subjectivity with estimation of deadweight and attribution (34, 35).

As suggested by this study, while not without its limitations, SROI analysis can be a useful methodology to practitioners, donors, researchers, and policy-makers who are interested in holistic measurement of impact, including value-for-money, of public health interventions. This study is, to authors knowledge, one of a handful to apply the social return on investment methodology to vision health, mobile health and child health interventions. This study demonstrates that through the direct involvement of stakeholders throughout the SROI process, a better quality of analysis can be achieved compared to traditional methods. Ultimately, the goal of program evaluation is to understand the strengths and weaknesses of the program of interest and provide recommendations to improve the program in order to better serve communities. Traditional economic evaluation methods in public health rely heavily on the insights provided by those conducting the analysis – which extends to the strengths and opportunities of the program, potentially missing key opportunities. Through the heavy involvement of stakeholders in the SROI process, direct feedback from these groups can be collected – in turn, informing the final analysis, results, and recommendations. Such indicators and insights are often missed by traditional economic approaches used in the public health field. If ultimately, what gets measured, gets valued, it is of prime importance to ensure we are measuring all that truly matters to all of those who are affected by an intervention to prove and improve the value of public health interventions.

## Data Availability

The raw data supporting the conclusions of this article will be made available by the authors, without undue reservation.

## References

[1] Masters R Anwar E Collins B Cookson R Capewell S. Return on investment of public health interventions: a systematic review. J Epidemiol Community Health. (2017) 71:827–34. doi: 10.1136/jech-2016-208141, 28356325 PMC5537512

[2] Muir Gray JA. Better value healthcare – the 21st century agenda. Zeitschrift für ärztliche Fortbildung und Qualität im Gesundheitswesen – German Journal for Quality in Health Care. (2007) 101:344–6. doi: 10.1016/j.zgesun.2007.04.009, 17711262

[3] Putera I. Redefining health: implication for value-based healthcare reform. Cureus. (2017) 9:e1067. doi: 10.7759/cureus.1067 38348426 PMC10860730

[4] Vermaire JH van Loveren C Brouwer WBF Krol M. Value for money: economic evaluation of two different caries prevention programmes compared with standard Care in a Randomized Controlled Trial. Caries Res. (2014) 48:244–53. doi: 10.1159/000356859, 24526078

[5] Banke-Thomas AO Madaj B Charles A van den Broek N. Social return on investment (SROI) methodology to account for value for money of public health interventions: a systematic review. BMC Public Health. (2015) 15:582. doi: 10.1186/s12889-015-1935-7, 26099274 PMC4477315

[6] Vogel LH. Finding value from IT investments: exploring the elusive ROI in healthcare. J Healthc Inf Manag. (2003) 17:20–8. 14558368

[7] Dolan P Edlin R. Is it really possible to build a bridge between cost-benefit analysis and cost-effectiveness analysis? J Health Econ. (2002) 21:827–43. doi: 10.1016/S0167-6296(02)00011-5 12349884

[8] Zarnke K Levine M O’Brien B. Cost-benefit analyses in the health-care literature: don't judge a study by its label. J Clin Epidemiol. (1997) 50:813–22. doi: 10.1016/S0895-4356(97)00064-4 9253393

[9] Banke-Thomas A Madaj B Kumar S Ameh C van den Broek N. Assessing value-for-money in maternal and newborn health. BMJ Glob Health. (2017) 2:e000310. doi: 10.1136/bmjgh-2017-000310, 29081998 PMC5656121

[10] Kumar S Banke-Thomas A. Social return on investment (SROI): An innovative approach to sustainable development goals for sexual and reproductive health programming in sub-Saharan Africa. Afr J Reprod Health. (2016) 20:85–93. doi: 10.29063/ajrh2016/v20i3.13 29553198

[11] Social Value International. The purpose of the principles of social value and the SVI standards. (2022). London: Social Value International.

[12] Lingane A Olsen S. Guidelines for social return on investment. Calif Manag Rev. (2004) 46:116–35. doi: 10.2307/41166224

[13] Social Value International. A guide to social return on investment. (2012). London: Social Value International.

[14] Dale N Salt A. Early support developmental journal for children with visual impairment: the case for a new developmental framework for early intervention. Child Care Health Dev. (2007) 33:684–90. doi: 10.1111/j.1365-2214.2007.00798.x 17944777

[15] Burgmeier R Desai RU Farner KC Tiano B Lacey R Volpe NJ . The effect of amblyopia on visual-auditory speech perception: why mothers may say “look at me when i’m talking to you.”. JAMA Ophthalmol. (2015) 133:11–6. doi: 10.1001/jamaophthalmol.2014.3307 25211190

[16] Ayaki M Torii H Tsubota K Negishi K. Decreased sleep quality in high myopia children. Sci Rep. (2016) 6:33902. doi: 10.1038/srep33902 27650408 PMC5030671

[17] Basch CE. Vision and the achievement gap among urban minority youth. J Sch Health. (2011) 81:599–605. doi: 10.1111/j.1746-1561.2011.00633.x, 21923871

[18] Mathers M Keyes M Wright M. A review of the evidence on the effectiveness of children’s vision screening: effectiveness of children’s vision screening. Child Care Health Dev. (2010) 36:756–80. doi: 10.1111/j.1365-2214.2010.01109.x, 20645997

[19] Glewwe P West KL Lee J. The impact of providing vision screening and free eyeglasses on academic outcomes: evidence from a randomized trial in title I elementary schools in Florida: impact of providing vision screening and eyeglasses on academic outcomes. J Policy Anal Manage. (2018) 37:265–300. doi: 10.1002/pam.22043, 29693366 PMC5959017

[20] Belli P Bustreo F Preker A. Investing in children’s health: what are the economic benefits? Bull World Health Organ. (2005) 83:777–84. 16283055 PMC2626422

[21] Social Value International (n.d.). Principle 5: Do Not Overclaim. Available at: https://www.socialvalueint.org/principle-5-do-not-overclaim (Accessed July 19, 2024).

[22] Wittenborn JS Zhang X Feagan CW Crouse WL Shrestha S Kemper AR . The economic burden of vision loss and eye disorders among the United States population younger than 40 years. Ophthalmology. (2013) 120:1728–35. doi: 10.1016/j.ophtha.2013.01.068, 23631946 PMC5304763

[23] McGrath R Stevens K. Cirkidz youth circus skills training social return on investment (SROI) report 2017. (2017). Adelaide: University of South Australia.

[24] Lakhotia S Te Whānau o Waipareira Trust Ngā tau Mīharo o Aotearoa: Incredible years parenting programme social impact report, Auckland (2019).

[25] Ernst and Young, Eastern Media International, Eastern Charity Foundation. (2020). Social Return on Investment (SROI) Report on the Love Breakfast Project. Available at: https://socialvalueuk.org/wp-content/uploads/2021/02/SROI-Report-on-Love-Breakfast-Project_vol.6_0217.pdf (Accessed August 12, 2021).

[26] Joish V Malone D Miller J. A cost-benefit analysis of vision screening methods for preschoolers and school-age children. J AAPOS. (2003, 2003) 7:12917617:283–90. doi: 10.1016/s1091-8531(03)00116-2, 12917617

[27] White A Abt Associates (2004). Eye exams for children: their impact and cost effectiveness. Report prepared for the Vision Council of America. Available at: https://www.abtglobal.com/sites/default/files/files/Insights/reports/2004/Cost_Effectiveness_of_Eye_Exams.pdf

[28] Ebri AE Govender P Naidoo K . Understanding barriers to spectacle wear compliance among schoolchildren in Calabar Nigeria: a qualitative study. AJO Int. (2025). 2. doi: 10.1016/j.ajoint.2025.100160

[29] Kovai V Krishnaiah S Shamanna BR Thomas R Rao GN. Barriers to accessing eye care services among visually impaired populations in rural Andhra Pradesh, South India. Indian J Ophthalmol. (2007) 55:365–71. doi: 10.4103/0301-4738.33823, 17699946 PMC2636013

[30] Morjaria P McCormick I Gilbert C. Compliance and predictors of spectacle Wear in schoolchildren and reasons for non-Wear: a review of the literature. Ophthalmic Epidemiol. (2019) 26:367–77. doi: 10.1080/09286586.2019.1628282, 31181970

[31] Wu L Feng J Zhang M. Implementing interventions to promote spectacle wearing among children with refractive errors: a systematic review and meta-analysis. Front Public Health 2023;11:1053206. Published 2023 Mar 10. doi: 10.3389/fpubh.2023.1053206, 36969641 PMC10036364

[32] Arvidson M Lyon F McKay S Moro D. The ambitions and challenges of SROI. Birmingham: Third Sector Research Centre, University of Birmingham (2010).

[33] Edwards RT Lawrence CL. ‘What you see is all there is’: the importance of heuristics in cost-benefit analysis (CBA) and social return on investment (SROI) in the evaluation of public health interventions. Appl Health Econ Health Policy. (2021) 19:653–64. doi: 10.1007/s40258-021-00653-5, 34056701 PMC8164934

[34] Corvo L Pastore L Mastrodascio M Cepiku D. The social return on investment model: a systematic literature review. Meditari Account Res. (2022, 2022) 30:49–86. doi: 10.1108/MEDAR-05-2021-1307

[35] Gosselin V Boccanfuso D Laberge S. Social return on investment (SROI) method to evaluate physical activity and sport interventions: a systematic review. Int J Behav Nutr Phys Act. (2020) 17:26. doi: 10.1186/s12966-020-00931-w, 32106872 PMC7047368

