# Supplementary material for: A holistic approach for quantifying the value of public health programs: social return on investment (SROI) analysis of a mobile clinic as an example
Source: Front Public Health. 2026 Feb 25;14:1650485. doi: 10.3389/fpubh.2026.1650485 (PMC12975873; doi:10.3389/fpubh.2026.1650485)
Supplement: Supplementary file 1 [file Data_Sheet_1.docx]

## **Appendix. Guide for Survey Interviews with Parents.**

Hi, my name is [name of interviewer], I am calling because you have a son/daughter that received an eye exam at the UCLA mobile eye clinic in the year of [year]. We are conducting interviews of families to learn how this exam has affected your son/daughter. We value your comments and opinions which is important for us to learn the effects of the program and how we can improve the UCLA mobile eye clinic in the future.

The purpose of this interview is to learn about UMEC screening and exam so we can improve it. There are no right or wrong answers, and anything you say is helpful. This will not affect your child’s access to the service.

First, I’m going to ask a few things about you so we know whether the program is reaching everyone who might benefit from it.

[Prefill these questions]:

1. What part of LA do you live in? (use UCLA standard set of options)
2. Is your child a boy or girl? (Boy/girl)
3. In what year was he/she screened? (2012-2017)

Screening and Logistics:

1. Do you recall your child being screened by the UCLA mobile eye clinic? (Yes/no/not sure) [If no or not sure, remind them what the process was]
2. Do you have more than one child who was screened? (Yes/no/not sure)
3. Were you invited in advance by the program to attend? (Yes, and I attended/Yes, but I did not attend / Don’t remember / No)
4. What time of day did the screening occur, if you recall? (AM/Mid-day/PM)
5. Was it at a time you could attend? (Yes/no)
6. Is there a time that might work better? (6-8am, 8-10am, 10-12pm, 12-2pm, 2-4pm, 4-6pm, No)
7. Was the screening at a location where you could attend? (Yes/no)

Family History:

1. Does anyone in your child’s family wear glasses? (Yes/no)
2. Did you have glasses as a child? (yes/no)
3. Do you have glasses or contacts now? (yes/no/no, corrective surgery)
4. Does the child’s other parent wear glasses? (yes/no)

Regular doctor:

1. Now, thinking about your child’s regular doctor, had your pediatrician ever done an eye test of any kind for your child? (Yes/no/not sure)
2. If so, did your child’s doctor/pediatrician say glasses were needed, or were not needed? (yes needed, not needed, not sure)
3. Did the UMEC screening show that glasses were recommended? (Yes/no/don’t remember) And if yes, did your child receive glasses from UMEC? (yes/no)
   1. If UMEC said that glasses were needed, but your pediatrician said they were not needed, did your pediatrician tell you they were not needed before or after the UMEC screening? (Before UCLA screening; After UCLA screening; Both)
   2. If UMEC said glasses were needed, but your pediatrician said they were not needed, did your pediatrician’s advice affect whether you got the glasses prescription filled? (Yes, no)
   3. Do you think the UMEC eye screening influenced your decision to get glasses? (Not at all, somewhat, very much)

ALTERNATE SCENARIO 1: If told by the UMEC screening they didn’t need glasses:

1. Did you notice any immediate changes after the screening in your child’s ability to read, or play, or anything else? (yes/no)
   1. If yes, on a scale of 1-10 how much of this change is due to the UCLA eye screening? (1-10)
2. Does the child have glasses now?
3. How much do you think the UCLA eye screening played a role in you finding out your child’s need for eye care? (major role, minor role, no role)
4. Is there anything you wish the program did differently?
5. Is there anything you wish the program did that it doesn’t currently do?

ALTERNATE SCENARIO 2: If told by UMEC screening they did need glasses, but the glasses never made it to them from UMEC:

1. Did you notice any immediate changes after the screening in your child’s ability to read, or play, or anything else? (yes/no)
   1. If yes, on a scale of 1-10 how much of this change is due to the UCLA eye screening? (1-10)
2. Does the child have glasses now?
3. How much do you think the UCLA eye screening played a role in you finding out your child’s need for eye care? (major role, minor role, no role)
4. Is there anything you wish the program did differently?
5. Is there anything you wish the program did that it doesn’t currently do?

ALTERNATE SCENARIO 3: If told by UMEC screening they need glasses and they received glasses from UMEC:

1. What changed right away for your child as a result of the exam/glasses? (open-ended question)?
   1. Since receiving glasses, did you notice differences in your child’s ability to see/quality of life? (Yes/somewhat/no/unsure)
   2. Since receiving glasses, did you notice differences in your child’s attentiveness in school? (Yes/somewhat/no/unsure)
   3. Since receiving glasses, do you feel that your child’s participation or play was different? (Yes/somewhat/no/unsure)
   4. Since receiving glasses, do you feel that your child’s dependence on others was different? (Yes/somewhat/no/unsure)
   5. Since receiving glasses, was your child’s self-confidence different? (Yes/somewhat/no/unsure)
   6. Before/after receiving glasses, does your child have good self-esteem or confidence? (on a 1-5 Likert scale, 1 being strong no, 5 being strong yes)
   7. Since receiving glasses, does your child feel more responsible? (on a 1-5 Likert scale, 1 being strong no, 5 being strong yes)
   8. Before/after receiving glasses, did your child ever have headaches? (Yes/somewhat/no/unsure)
   9. Before/after receiving glasses, did your child experience depression? (Yes, no)
   10. Before/after receiving glasses, did your child have any trouble sleeping? (Yes, somewhat, no)
   11. Before/after receiving glasses, had your child received any sort of behavioral diagnosis (e.g., ADHD, autism, or any other?) (Yes/no/unsure)
   12. Before/after receiving glasses, how would you describe your child’s attentiveness at school? (Very attentive, somewhat attentive, inattentive, unsure)
   13. Before/after receiving glasses, what is your child’s average rating in school? (O, S, or N – outstanding, satisfactory, needs improvement, or unsure)
   14. Before/after receiving glasses, is your child performing above, at or below grade level? (above, at, below, unsure)
   15. Before/after receiving glasses, does your child feel socially isolated (Likert scale 1-5; 1 being strong no and 5 being strong yes)?
   16. Before/after receiving glasses, does your child have a reputation for being clumsy (Likert scale 1-5; 1 being strong no and 5 being strong yes)?
   17. Before/after receiving glasses, does your child have behavioral incidents that cause school/teacher to intervene or contact parents? (never, very rarely, once/month, once/daily)
   18. Before/after receiving glasses, does your child experience teasing or bullying? (very often, occasionally, none, unsure)
2. [For each of 1-3 major outcomes above] How important was [each change above] to the child? (on a scale of 1-5)
3. [For each major outcome above] How important was [each change above] to you as the parent/guardian? (on a scale of 1-5)
4. For each outcome/change above, has the change persisted? For how long? (less than 1 year, 1 year, 2 years, 3 years, 4 years, 5 or more years, unsure)
5. How much of the changes that you observed in your child as a result of the screening/glasses were contributed to by other people or organizations? For example: a family member helping the child with reading outside of school, or a therapist contributing to the child's sense of well-being?
   1. What percentage of the change would you say was attributable to others than UMEC? (none (0%), <25%, 26-50%, 51-75%, >75%, not sure)
6. Is there anything you wish the program did differently?
7. Is there anything you wish the program did that it doesn’t currently do?

Thank you so much for your time!
